# An outbreak of HIV infection among people who inject drugs in northeastern Massachusetts: findings and lessons learned from a medical record review

**DOI:** 10.1186/s12889-022-12604-3

**Published:** 2022-02-08

**Authors:** Liisa M. Randall, Sharoda Dasgupta, Jeanne Day, Alfred DeMaria, Joseph Musolino, Betsey John, Kevin Cranston, Kate Buchacz

**Affiliations:** 1 Massachusetts Department of Public Health, Bureau of Infectious Disease and Laboratory Sciences, 305 South Street, Jamaica Plain, MA 02130-3515 USA; 2 Division of HIV/AIDS Prevention, Centers for Disease Control and Prevention, Atlanta, GA USA; 3 JSI Research and Training Institute, Inc., Boston, MA USA

**Keywords:** HIV, HIV outbreak, Massachusetts, Medical records, PWID, Health care, Care continuity

## Abstract

**Background:**

We conducted a medical record review for healthcare utilization, risk factors, and clinical data among people who inject drugs (PWID) in Massachusetts to aid HIV outbreak response decision-making and strengthen public health practice.

**Setting:**

Two large community health centers (CHCs) that provide HIV and related services in northeastern Massachusetts.

**Methods:**

Between May and July 2018, we reviewed medical records for 88 people with HIV (PWH) connected to the outbreak. The review period included care received from May 1, 2016, through the date of review. Surveillance data were used to establish date of HIV diagnosis and assess viral suppression.

**Results:**

Sixty-nine (78%) people had HIV infection diagnosed during the review period, including 10 acute infections. Persons had a median of 3 primary care visits after HIV diagnosis and zero before diagnosis. During the review period, 72% reported active drug or alcohol use, 62% were prescribed medication assisted treatment, and 41% were prescribed antidepressants. The majority (68, 77%) had a documented ART prescription. HIV viral suppression at < 200 copies/mL was more frequent (73%) than the overall across the State (65%); it did not correlate with any of the sociodemographic characteristics studied in our population. Over half (57%) had been hospitalized at least once during the review period, and 36% had a bacterial infection at hospitalization.

**Conclusions:**

Medical record review with a field investigation of an outbreak provided data about patterns of health care utilization and comorbidities not available from routine HIV surveillance or case interviews. Integration of HIV screening with treatment for HIV and SUD can strengthen prevention and care services for PWID in northeastern Massachusetts.

## Introduction

Worldwide, 9% of new HIV infections are attributed to injection drug use (IDU) [1]. In the United States (U.S.), 6% of diagnoses of HIV infection are attributed IDU [2]. Persons who inject drugs (PWID) are at risk for HIV infection through sharing of non-sterile injection equipment and sexual behaviors [3, 4]. Over the past decade, multiple HIV outbreaks have occurred among PWID in high and middle-income countries, including in North America and Europe [5]. Commonalities of outbreaks include shifting drug use patterns, extensive homelessness, history of incarceration, and co-occurrence of hepatitis C infection. Some areas that experienced outbreaks were generally well-covered by harm reduction and other health services [6–11]. Globally, it is estimated that 15 million people use injection drugs, among whom approximately 22% have experienced recent homelessness, 58% have history of incarceration, and 18% are living with HIV [12]. In the context of the opioid crisis in the US, increasing frequency of IDU and fentanyl use may increase HIV transmission risk among PWID [13, 14]. This has been documented in several HIV outbreaks around the U.S. [15–17].

Massachusetts has been hard hit by the opioid epidemic; by 2015 there were 1526 annual opioid-related deaths [18]. Nonetheless, between 2000 and 2014, the number of reported HIV infections in Massachusetts declined by 47% overall and by 91% among PWID [19]. In August 2016, 5 new HIV diagnoses were reported to the Massachusetts Department of Public Health (MDPH) among PWID in Lawrence, Massachusetts. This was an unexpectedly large number of diagnoses in this city, given an average of 1 new HIV diagnosis per month in the preceding two years. Subsequently, MDPH initiated investigation and enhanced response efforts in collaboration with local stakeholders. Investigation identified additional HIV cases among PWID linked to Lawrence, and to nearby Lowell, Massachusetts. Epidemiologic investigation and qualitative interviews suggested sharing of syringes and other injection equipment was frequent among individuals involved in the cluster. Homelessness, history of incarceration, and exchanging sex for drugs was also commonly reported. Investigation indicated that fentanyl had replaced heroin in these communities, resulting in increased frequency of injection [20]. By June of 2019, 166 persons were linked to the outbreak [21].

MDPH, in collaboration with the U.S. Centers for Disease Control and Prevention (CDC), conducted an investigation to characterize the outbreak, evaluate precipitating factors, and identify appropriate interventions between April and June 2018. Alpern et al. present a detailed timeline of the outbreak and subsequent investigation﻿﻿ [21]. Investigation strategies included patient interviews by public health epidemiologists, molecular analysis of HIV genetic sequences, qualitative interviews with PWID and stakeholders, and review of medical records for persons associated with the outbreak. This paper focuses on the medical records review.

During an HIV outbreak and response, public health authorities and collaborating community and federal partners are challenged to quickly gather relevant multifaceted data describing the public health problem and affected population, and to identify and implement strategies that arrest HIV transmission. Our goal in performing an enhanced medical records review was to supplement surveillance and epidemiologic data by ascertaining medical care engagement and potential gaps in care, assessing substance use and treatment, and evaluating comorbidities and outcomes among persons identified in the HIV outbreak, before and after HIV diagnosis. A deeper understanding of patterns in medical care and comorbidities was needed to effectively approach improving health outcomes and preventing HIV transmission among PWID in northeastern Massachusetts.

## Methods

### Setting and sites

MDPH leveraged a routine HIV medical record review, at two community health centers (CHCs), the Greater Lawrence Family Health Center, and the Lowell Community Health Center. These CHCs receive state and federal funding for HIV services, they are key HIV care providers in their communities, and collaborated in the outbreak investigation. Both facilities exchange patient information with affiliated hospitals and other facilities in their communities, and information about hospital admissions, emergency department visits, and substance use disorder (SUD) treatment was abstracted whenever available in records.

### Eligibility

A total of 138 individuals with HIV infection, who were under investigation as linked to the outbreak, were eligible for medical record review as of May 15, 2018. An outbreak case was defined as a person diagnosed with HIV infection in Massachusetts during January 2015 to May 2018 and who received medical care, experienced homelessness, resided, or injected drugs in Lawrence or Lowell; was epidemiologically linked as an injecting or sex partner of a person with HIV infection connected to Lawrence or Lowell; or had an HIV-1 *pol* nucleotide sequence molecularly linked at a genetic distance of ≤1.5% to that of another person in the investigation. Eligibility for medical record review was further limited to persons who received medical care at the participating CHCs.

### Data collection and sources

A chart abstraction tool used by MDPH for routine HIV medical record review was expanded and included: patient demographics and social history; HIV risk exposure; medical visits, including dates, reason for and type of visit (primary care, acute/urgent care, and emergency); screening for HIV infection; HIV treatment, including diagnosis date, HIV viral load and CD4 cell count test results, and antiretroviral treatment (ART); screening for and treatment of sexually transmitted infections (STIs) and hepatitis C (HCV); hepatitis B (HBV) immunity; history of SUD and treatment, including medication assisted treatment (MAT); and hospitalizations, including dates, facility, reasons for admission, and diagnosis of bacterial infection. Data were abstracted from medical records by trained and experienced medical record abstractors. MDPH, through collaboration with JSI Research and Training (JSI), has conducted routine review of medical records since 1999.

Reviews were conducted by JSI staff between May 17 and June 6, 2018 for outbreak-linked patients who had evidence of care at the participating CHCs. The review period was for care received from May 1, 2016 through the date on which the review was performed. Data were recorded on paper forms, and manually entered into a database. Quality assurance identified missing and out-of-range values. Abstracted data were combined with routine HIV surveillance data as of October 31, 2018 to ascertain the date of first reported laboratory evidence of HIV infection, and to obtain HIV viral load results for tests performed but not captured in the records. In case of discrepancies in the date of HIV diagnosis, surveillance data were used. In Massachusetts, HIV surveillance data includes results from all laboratory testing within the state, thereby providing more complete information regarding diagnosis and viral suppression status.

### Variable definitions

Engagement in care was defined based on documentation of primary care visits during the review period. Viral suppression was defined as a viral load (VL) of < 200 copies per mL, at the last recorded test; and a VL of < 20 copies per mL at the last recorded test was considered an undetectable VL. Acute HIV infection was defined by a positive HIV RNA nucleic acid test result subsequent to a positive HIV antigen/antibody screening result with a negative or indeterminate supplemental antibody test. Patients were considered “on ART” if there was documentation of an ART prescription in the medical record at the most recent medical visit. History of HCV infection was based on positive HCV antibody or RNA results, or documented diagnosis. Hepatitis B infection or immunity was established based on laboratory test results for surface antigen, surface antibody, core antibody, or HBV DNA positivity. Patients were classified as having had an STI if there was documentation of one or more instances of diagnosis with syphilis, gonorrhea, or chlamydia at any time during the review period. Patients were considered to have been incarcerated, homelessness, or have had drug or alcohol use history if any instance, regardless of duration, was documented in the record during the review period.

### Analyses

We performed descriptive analyses of demographic, social history, and clinical care utilization characteristics for the cohort. We also examined healthcare utilization (types and number of medical encounters) before and after HIV diagnosis during the review period, and stratified according to whether patients achieved viral suppression at the most recent VL measurement during the review period, or the 4 months thereafter based on surveillance data. Patients had variable observation time and opportunity for HIV care engagement after HIV diagnosis and during the review period. The two-tailed Fisher’s exact chi-square test was used to evaluate differences between groups, and results with *P* < 0.05 were considered statistically significant.

## Results

### Patient population

A total of 138 individuals with HIV infection were initially linked to the outbreak as of May 15, 2018. Of these, 29 (21%) had no indication of engagement in medical care at either CHC and 21 (15%) were patients of record at one of the CHCs, but had no documented medical visit during the review period. The medical record review was limited to 88 (81%) of the 109 patients of record at one or both CHCs (11 persons had records at both CHCs). When comparing the characteristics of the 88 patients included and 50 patients excluded, those included were more likely to have HIV diagnoses in 2017 or later (65% versus 42%, *p* = 0.01). Demographic differences were modest and not statistically significant (data not shown).

### Demographic characteristics

Of the 88 patients whose records were reviewed (Table 1), 54 (61%) were male; 46 (52%) were white, non-Hispanic; 61 (70%) under 40 years of age; and 71 (81%) reported IDU, as a sole exposure risk for HIV infection. During the review period, 19 (22%) persons had documentation of incarceration, and 65 (74%) had documentation of homelessness. A majority (*n* = 73, 83% had Medicaid and/or Medicare.

**Table 1:** Sociodemographic characteristics of patients with HIV infection whose medical records were reviewed, northeast Massachusetts, 2018 (*N* = 88)

Sociodemographic Characteristic	n	%
Sex		
Male	54	61
Female	34	39
Race/Ethnicity		
White non-Hispanic	46	52
Black non-Hispanic	4	5
Hispanic/Latino	33	38
Other/unknown	5	6
Age (as of 6/30/2018, years)		
20-29	26	30
30-39	35	40
40-49	15	17
50-59	7	8
> 60	5	6
Place of birth		
United States mainland	50	57
Puerto Rico	18	21
Other	3	3
Unknown	17	19
HIV risk exposure		
Male to male sexual contact (MSM)	0	0
Heterosexual only	12	14
Injection drug use (IDU)	71	81
MSM/IDU	1	1
Unknown/not documented	4	5
Year of HIV diagnosis^b^		
Prior to 2015	11	13
2015	5	6
2016	15	17
2017	46	52
2018	11	13
Age (at HIV diagnosis, years)^b^		
< 19	1	1
20-29	29	33
30-39	35	40
40-49	14	16
50-59	6	7
> 60	3	3
Incarcerated during review period^a^		
Yes	19	22
No	69	78
Homeless during review period^a^		
Yes	65	74
No	20	23
Not Documented	3	3
Health insurance coverage (as of 6/30/18)		
Medicare	10	11
Medicaid	72	82
Medicare/Medicaid	1	1
Private health insurance	1	1
None, self-pay	4	5

^a^As determined through medical record review, and HIV surveillance data reported to the Massachusetts Department of Public Health through October 31, 2018. Medical record review period was May 1, 2016 through date of data abstraction (abstraction performed May 17 to June 6, 2018)

^b^As documented HIV surveillance data reported to the Massachusetts Department of Public Health

### Substance use history

A majority (*n* = 63, 72%) of patients had documentation of active drug or alcohol use during the review period, with 79% reporting use of heroin, 54% cocaine, 37% other opiates, and 16% alcohol (Table 2). Among those reporting active drug use, injection was the primary method of consumption for (*n* = 59, 94%). A majority (*n* = 39, 62%) of patients documented with active drug or alcohol use had documentation of MAT during the review period, and 23 (37%) patients had documentation of receipt of other treatment modalities (Table 2).

**Table 2:** Drug and alcohol use and treatment among patients with HIV infection whose medical records were reviewed, northeast Massachusetts, 2018 (*N* = 88)

	n^a^	%
Drug alcohol and drug use during the review period		
Active use in review period	63	72
History of use/inactive in review period	8	9
History of use/inactive in review period, on suboxone	11	13
No history	5	6
Missing	1	1
Among those with documented active drug or alcohol use (*n* = 63), substances used (categories not mutually exclusive)		
Alcohol	10	16
Cocaine	34	54
Heroin	50	79
Other opiates	23	37
Methamphetamine	1	2
Recreational use of prescription drugs	1	2
Not documented	3	5
Other^b^	8	13
Route of drug use (*n* = 63) (categories not mutually exclusive)		
Injection	59	94
Intranasal	9	14
Smoking	7	11
Oral	9	14
Not Documented	3	5
Treatment for drug/alcohol use for active substance users (*n* = 63), modality (categories not mutually exclusive)		
Medication assisted treatment	39	62
In treatment, another modality^c^	23	37
Counseled by provider	15	24
No action indicated	4	6
Among those reporting another modality of treatment (*n* = 23), modality (categories not mutually exclusive)		
Counseling	9	39
Twelve-step program	3	13
Halfway/sober house	9	39
Other	10	43

^a^Medical record review period was May 1, 2016 through date of data abstraction (abstraction done May 17 to June 6, 2018), and all characteristics were assessed during the review period

^b^Other active substances include suboxone/fentanyl (*n* = 1), methadone/fentanyl (*n* = 1), bath salts (*n* = 1), benzodiazepine (*n* = 2), marijuana (*n* = 3)

^c^Includes dedicated drug or alcohol counseling program, 12-step program, halfway/sober house, and other [detox program (*n* = 6), referral made (*n* = 3), and residential program (*n* = 1)]

### HIV related and clinical history

Overall, 69 (78%) patients had HIV infection diagnosed during the review period. Ten (11% of total) were diagnosed with acute HIV infection (Table 3). Among patients whose records were reviewed, 81 (92%) had documentation of one or more clinical encounters on or after the date of HIV diagnosis. All had documentation of a primary care visit during at least one of the 2 years reviewed, and 50 (57%) had a primary care visit documented in each of the 2 years. Over three-quarters (*n* = 68, 77%) of patients were prescribed ART at any time during the review period including 48 (55%) who initiated ART for the first time. Nearly all patients (*n* = 84, 95%) had at least one VL result on record by the end of October 2018. A majority (*n* = 61, 73%) of these 84 patients achieved viral suppression (< 200 copies/mL), and of these 50 (60%) were undetectable (< 20 copies/mL) at the last VL result.

**Table 3:** Clinical characteristics and engagement in care of patients with HIV infection whose medical records were reviewed, northeast Massachusetts, 2018 (*N* = 88)

	n^a^	%
Time since HIV infection diagnosis (as of June 30, 2018)		
< 6 months	13	15
6 months to one year	29	33
1- < 2 years	23	26
2- < 5 years	18	20
5+ years	5	6
Acute HIV infection documented during review period	10	11
In care since HIV diagnosis (one or more medical visits on or after date of diagnosis)		
Yes	81	92
No	7	8
In care at CHC during review period (had at least 1 primary care visit in each review year)		
In care both years	50	57
In care 2016/2017 only	8	9
In care 2017/2018 only	30	34
On anti-retroviral therapy (ART) during review period		
Yes	68	77
No	20	23
Initiated ART for the first time during the review period		
Yes	48	55
No	40	45
Viral load (VL) test results during the review period^b^		
> 1 VL test	84	95
At least 2 VL tests	69	78
No VL test	4	5
Viral load suppression (< 200 copies/mL)^b^ (*n* = 84)		
Most recent VL suppressed	61	73
Most recent VL unsuppressed	23	27
Viral load below limit of detection (< 20 copies/mL)^b^ (*n* = 84)		
Most recent VL undetectable	50	60
Most recent VL detectable	34	40
Hospitalized during the review period		
Yes	50	57
No	38	43
Bacterial infection among hospitalized (*N* = 50)		
Yes	18	36
No	32	64
Bacterial infections among hospitalized (*N* = 18) (categories not mutually exclusive)		
Bacteremia	8	44
Endocarditis	2	11
Osteomyelitis	0	0
Cellulitis	11	61
Other	3	17
Hepatitis C infection^c^		
History documented (ever)	72	82
Treatment documented (ever)	5	7
Hepatitis B immune status^c^ (through end of review period)		
Positive	58	66
Negative	22	25
Unknown	8	9
If Hepatitis B negative or unknown (*n* = 30), received 3 HBV vaccine doses		
Yes	8	27
No	22	73
Sexually transmitted infections during review period^d^		
Yes	8	9
No	80	91
Prescribed any of the following medications during the review period		
Any Opioid	5	6
Fentanyl	0	0
Oxycodone	0	0
Methadone	26	30
Naltrexone	13	15
Buprenorphine	0	0
Buprenorphine/naloxone	22	25
Benzodiazepine or another anxiolytic	7	8
Antidepressant	36	41
Antipsychotic	18	21

^a^As determined through medical record review, and HIV surveillance data (for viral load measures, only) reported to the Massachusetts Department of Public Health through October 31, 2018. Medical record review period was May 1, 2016 through date of data abstraction (abstraction done May 17 to June 6, 2018)

^b^Based on information from chart review supplemented with HIV surveillance data

^c^Hepatitis B immune status definitions: positive: if positive result for surface antigen, surface antibody, core antibody, or presence of HBV DNA; negative: if negative result for both surface antibody and core antibody or HBV DNA negative

^d^Diagnosed sexually transmitted infections include gonorrhea (*n* = 3), chlamydia (*n* = 3), and syphilis (*n* = 2)

Over half (*n* = 50, 57%) of patients had been hospitalized at least once during the review period. Of these, 18 (36%) had one or more bacterial infections. The most common bacterial infection was cellulitis (61%) followed by bacteremia (44%). Seventy-two (82%) patients had a history of HCV infection, but only five (7%) patients had documentation of ever receiving HCV treatment. Fifty-eight (66%) patients had evidence of current or past HBV infection or immunity, among the 30 (34%) without evidence of HBV infection or immunity (22) and without screening documented (8), only 10 (33%) had documentation of any HBV vaccine; 8 (27%) had received all three doses. An STI (syphilis, gonorrhea, or chlamydia) was diagnosed among eight (9%) patients during the review period. Documented prescriptions to treat mental health disorders included antidepressants (41%) and antipsychotics (21%). Documented MAT for opioid addiction included methadone (30%), buprenorphine/naloxone (25%), and naltrexone (15%) (Table 3).

### HIV viral load patterns and engagement in care

A significantly higher percentage of persons diagnosed prior to 2017 compared to those diagnosed in or after 2017 had viral suppression at the most recent measurement (Table 4). Similarly, a significantly higher percentage of patients with documented ART prescription were virally suppressed compared to those not prescribed ART (79% vs. 21%). No differences were found in viral suppression by sex, race/ethnicity, country of birth, HIV exposure risk, health insurance coverage, incarceration, homelessness, hospitalization, or MAT or other SUD treatment.

**Table 4:** Viral suppression (viral load < 200 copies per mL) by patient characteristics among patients with HIV infection whose medical records were reviewed and who had a reported viral load result, northeast Massachusetts, 2018 (*N* = 84)

	Overall^a^ (*N* = 84)	Most Recent VL Suppressed (*N* = 61)		Most recent VL Not Suppressed (*N* = 23)		*p* value^b^
	N	n	(row %)	n	(row %)	
Sex						
Male	53	38	72	15	28	1.00
Female	31	23	74	8	26	
Race/ethnicity						
White non-Hispanic	43	32	74	11	26	0.81
Black non-Hispanic	4	3	75	1	25	
Hispanic/Latino	33	23	70	10	30	0.63
Other/unknown	4	3	75	1	25	
Age (as of 6/30/2018, years)						
20-29	24	14	58	10	42	0.10
30-39	33	25	76	8	24	
40-49	15	12	80	3	20	
50-59	7	5	71	2	29	
≥ 60	5	5	100	0	0	
Place of birth						
United States	48	37	77	11	23	0.33
Puerto Rico	18	13	72	5	28	1.00
Other	3	2	67	1	33	
Unknown	15	9	60	6	40	0.34
HIV exposure risk						
Heterosexual only	12	10	83	2	17	0.50
IDU	67	47	70	20	30	0.38
MSM/IDU	1	0	0	1	100	
Unknown/not documented	4	4	100	0	0	0.57
Year HIV diagnosis						
Before and including 2016 ^c^	29	28	97	1	3	< 0.0001
2017-2018	55	33	60	22	40	
Health insurance coverage						
Medicare	9	7	78	2	22	1.00
Medicaid	69	50	72	19	28	
Medicare/Medicaid	1	0	0	1	100	
Private health insurance	1	1	100	0	0	
None, self-pay	4	3	75	1	25	
Incarceration during the review period						
Yes	19	15	79	4	21	0.57
No	65	46	71	19	29	
Homelessness during the review period						
Yes	63	45	71	18	29	0.78
No	18	14	78	4	22	
Not documented	3	2	67	1	33	
Hospitalizations during the review period						
Yes	49	36	73	13	27	1.00
No	35	25	71	10	29	
Medication assisted treatment prescribed during review period^d^						
Yes	50	37	74	13	26	0.81
No	34	24	71	10	29	
ART prescribed during review period						
Yes	68	54	79	14	21	0.01
No	16	7	44	9	56	
Substance use during review period (not mutually exclusive)	***n*** **= 83**	***n*** **= 60**		***n*** **= 23**		
Active use in review period	59	40	68	19	32	0.18
History of use/inactive in review period	8	6	75	2	25	
History of use/inactive in review period, on suboxone	11	9	82	2	18	
No history	5	5	100	0	0	
Treatment for drug and alcohol use (not mutually exclusive)	***n*** **= 59**	***n*** **= 40**		***n*** **= 19**		
Medication assisted treatment^d^	38	27	71	11	29	0.56
In treatment, another modality^e^	23	13	57	10	43	0.16
Counseled by provider	13	8	62	5	38	
No action indicated	3	2	67	1	33	
In treatment, another modality (not mutually exclusive)	***n*** **= 23**	***n*** **= 13**		***n*** **= 10**		
Counseling	9	4	44	5	56	0.42
12-step program	3	2	67	1	33	
Halfway/sober house	9	7	78	2	22	0.20
Other	10	4	40	6	60	

^a^As determined through medical record review, and HIV surveillance data (for viral load measures, only) reported to the Massachusetts Department of Public Health through October 31, 2018. Medical record review period was May 1, 2016 through date of data abstraction (abstraction done May 17 to June 6, 2018)

^b^Two-tailed Fisher Exact test *p*-value. Categories are compared to all other remaining categories unless otherwise indicated

^c^In analyses disaggregated by individual year of HIV diagnosis, HIV viral load suppression was 91% for 11 persons diagnosed before 2015, and was 100% for those diagnosed in either 2015 or 2016. It was 60% for those diagnosed in either 2017 or 2018. *P*-value applies to diagnoses before 2017 versus those during 2017-2018

^d^Includes naltrexone, buprenorphine/naloxone, and methadone

^e^Includes counseling, 12-step program, halfway/sober house, and other [detox program (*n* = 6), referral made (*n* = 3), and residential program (*n* = 1)]

We noted 71 patients (81%) had one or more instances of an unsuppressed VL; and 13 (15%) had documentation of viral suppression throughout the review period (data not shown). Among patients with unsuppressed VL, 18 (25%) had documented non-adherence to ART, and 46 (55%) had unsuppressed results at first measurement after HIV diagnosis.

Among the subset of 69 patients diagnosed with HIV infection during the review period, VL testing was done within 7 days of diagnosis in 15 patients (22%), within 30 days in 15 patients (22%), 30 to 90 days in 9 patients (13%), > 90 days in 26 patients (38%, range: 97 to 754 days, median: 189, mean: 264). Four (6%) patients had no reported viral load results. Forty-four (64%) of the 69 patients diagnosed during the review period achieved viral suppression (< 200 copies/mL) on their most recent VL measurement; 36 (52%) achieved an undetectable result (< 20 copies/mL). Of 49 patients with more than one VL measurement, 37 (76%) had achieved viral suppression while 30 (61%) achieved an undetectable viral load.

### Care utilization before and after HIV diagnosis

**Fig. 1:**
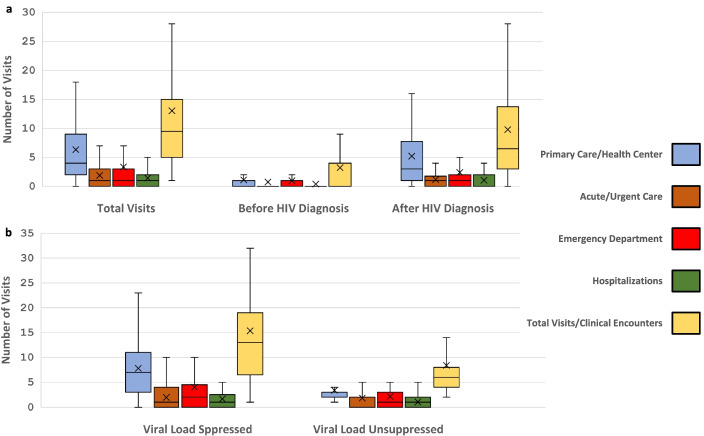
Box and whisker diagrams of number of visits/clinical encounters at clinical care sites documented on medical record review. Part **A**. All visits among the cohort of patients whose records were reviewed and visits before and after HIV infection diagnosis. Part **B**. Visits after diagnosis of HIV infection by achievement of viral suppression (< 200 copies per mL). Box = interquartile range (IQR), horizontal line = median, X = mean, whiskers represent 1.5 IQR, with outliers indicated

We examined the types and frequency of medical care encounters during the review period, before and after HIV diagnosis, and in relation to eventual viral suppression (Fig. 1). Patients had differing opportunities (i.e., available observation time) for medical care depending on whether they were diagnosed with HIV infection before the start of review period (*n* = 19) or during the review period (*n* = 69) and in relation to cut-off date for medical records abstraction. Before HIV diagnosis, evidence of clinical care was relatively limited; 47 (53%) had no medical visits or hospitalizations, and an additional 15 (17%) had no primary care visits. After HIV diagnosis, only seven (8%) patients had no visits or hospitalizations documented and an additional seven (8%) had no primary care visits. Patients who achieved viral suppression had greater engagement with clinical care, especially primary care, after HIV diagnosis, compared with patients who were not virally suppressed (Fig. 1).

## Discussion

PWID involved in this outbreak experienced a variety of structural and environmental conditions which contributed to HIV transmission risk, and impeded access to services and continuity of HIV care. Fentanyl use among PWID increased both the frequency of injection and the frequency of equipment sharing. Instability of housing, history of incarceration, and involvement in exchanging sex for drugs were common among PWID. While SSP services were operational in the two communities involved in the cluster, they were inadequate to respond to community need.

Medical record review was one component of a comprehensive field investigation and public health response to an HIV outbreak among PWID. Information gained through the review complemented epidemiologic analysis, routine public health interviews, qualitative interviews with PWID and community stakeholders, and molecular surveillance [21].

The findings from the medical record review suggest strengths and opportunities for improvement in the existing health care and public health systems, and strategies to enhance HIV prevention efforts for PWID. Specifically, we found limited use of health care services prior to HIV diagnosis, but extensive engagement after diagnosis, particularly in primary medical care. Emergency department use appeared to increase following diagnosis, suggestive of care for issues not well addressed in primary HIV care. We found evidence of marked engagement in drug and alcohol treatment, but possible suboptimal use of MAT. We documented high levels of ART prescription and viral suppression was higher than the overall rate in Massachusetts (65%), but lower than the state’s rate of viral suppression among patients retained in care (88%) [22]. Viral suppression correlated only with ART prescription and year of diagnosis.

Stakeholder interviews performed as part of the outbreak investigation [20], suggested that frequent homelessness and incarceration among PWID undermines HIV treatment continuity because of interrupted ART, missed appointments, and lack of coordination among care providers. However, we did not detect statistically significant differences in viral suppression by history of homelessness, incarceration, or by other sociodemographic characteristics. The combined efforts of public health and community providers may have mitigated challenges to HIV care continuity associated with these factors. Further exploration of how fragmentation of care affects care continuity and strategies to coordinate across the service system would increase understanding of assets and opportunities to ensure care continuity.

Stakeholder interviews [20] suggested active SUD as a barrier to HIV care engagement and adherence. With high levels of HIV ART and MAT prescription, our review suggests that concurrent treatment for HIV and SUD is feasible, and mutually reinforcing. Integration of screening and treatment for HIV and other infections (notably HCV infection) with opioid treatment, in various settings, should be prioritized as a strategy to engage PWID, deliver harm reduction services, facilitate early HIV diagnosis, and promote care continuity. Service integration may also facilitate identification and treatment of abscesses or wounds before they lead to conditions requiring hospitalization. At the same time, it is important to scale up harm reduction services for individuals who do not access SUD treatment. SSPs are effective in mitigating HIV risk by providing sterile injection equipment. The effectiveness of SSPs can be enhanced through provision of testing for HIV and other infections as well as other prevention services such as vaccinations and HIV PrEP [23].

MDPH used data from this medical record review, in combination with other knowledge generated through the outbreak investigation, to strengthen capacity to prevent and respond to HIV in Massachusetts including increasing public health capacity to follow-up on newly reported HIV infections; expanding the number of SSPs; and increasing availability of HIV testing services. MDPH implemented CME opportunities for primary care clinicians to increase understanding of the intersection of IDU and HIV, and to build knowledge necessary to screen for and treat HIV and other infections associated with drug injection. MDPH has also invested in training and technical assistance for SSPs to enhance the capability of SSP providers to provide HIV services, including testing, and linkage to HIV and SUD treatment. All training and technical assistance activities highlight barriers to care, and emphasize strategies to address missed opportunities for engagement in HIV services. There are a number of state and federal initiatives to strengthen integration of mental health and SUD treatment in CHCs, and to integrate SUD and HIV care. In Massachusetts, SSPs and correctional health programs are beginning to implement MAT.

Although MDPH has conducted medical record reviews to supplement routine HIV surveillance and monitor the quality of HIV care since 1999, this was the first time that record review was performed with an HIV outbreak investigation. As a result of this experience, an important lesson learned was that medical record review can substantially contribute to understanding social determinants of health and health status, including factors implicated in sub-optimal health outcomes. We gained insights about the frequency of urgent/acute care and hospitalization, bacterial infections, substance use, and engagement with SUD treatment. We found a high proportion of untreated HCV concurrent with HIV infection, prompting MDPH to intensify efforts to increase access to HCV treatment. The value of medical record review in responding to future outbreaks could be enhanced by developing a capacity and infrastructure that enables real-time, secure, electronic exchange of relevant clinical data between public health and clinical providers.

Our findings demonstrate that medical record review in the context of an outbreak investigation can benefit health jurisdictions without integrated infectious disease management systems by providing data on co-occurring conditions or clinical indications of early diagnosis. Medical records data can also suggest structural barriers to accessing services, missed opportunities for public health intervention and can facilitate exchange of information between surveillance and field staff needed to identify possible factors for transmission [24, 25]. Secondary benefits may include strengthening relationships between public health and clinical providers to improve efficiency in identifying and addressing emergent public health problems.

Our findings are subject to some limitations. While we were successful in abstracting records for a majority of individuals linked to this cluster, the overall sample size may have been insufficient to detect statistically significant differences in demographic factors examined. In addition, 50 individuals did not have records at either CHC, and their care patterns or clinical outcomes may be different from those of patients’ whose records we reviewed. For individuals whose records were reviewed, health care received outside of the networks of the participating CHCs and not captured in their medical records was not reviewed. Therefore, our understanding of HIV treatment and health care utilization of this cohort is incomplete. Some risk behaviors, including substance use, may not be reported or entered into the medical record, leading us to underestimate their frequency.

Massachusetts does not have city or county public health clinics. The state’s 52 CHCs function in a similar manner as public health clinics function in other U.S. States. Massachusetts is also distinctive in that almost all residents have health insurance, with less than 3% uninsured in 2017 [26]. As a result, public health in Massachusetts delivers HIV prevention, testing, and care coordination services through a robust network of CHCs, community-based organizations, and safety net hospitals. In this regard, the experience of Massachusetts may not reflect what other health jurisdictions face in the context of responding to an outbreak of similar magnitude.

## Conclusion

The combined and substantial response by public health and community providers in the face of an outbreak was, to a large degree, successful in engaging PWID in HIV treatment and promoting viral suppression. Ongoing, highly coordinated efforts to support care continuity must be sustained. A large percentage of the cohort had been treated for mental health disorders and had histories of homelessness, SUD treatment, hospitalizations for bacterial infections, and untreated HCV infection. This highlights the value of providing clinical and support services proximal to HIV medical care for PWID. It also underscores the importance of collaboration between public health and community providers to identify and intervene in transmission networks. Strengthening these collaborations will be essential to ending the HIV epidemic in Massachusetts and across the nation.

## Data Availability

The datasets generated and/or analyzed during the current project are not publicly available due to sensitive information contained in HIV surveillance and patient medical records. HIV surveillance data are subject to federal and state confidentiality laws. Minimal de-identified aggregate data in the form of tables are available from the corresponding author on reasonable request and subject to corresponding author’s institutional approval.

## Abbreviations

ART: Antiretroviral treatment
CDC: U.S. Centers for Disease Control and Prevention
CHC: Community health center
DNA: Deoxyribonucleic acid
HBV: Hepatitis B virus
HCV: Hepatitis C virus
HIV: Human immunodeficiency virus
IDU: Injection drug use
MAT: Medication assisted treatment
MDPH: Massachusetts Department of Public Health
PWID: Person(s) who inject drugs
RNA: Ribonucleic acid
STI: Sexually transmitted infection
SUD: Substance use disorder
U.S.: United States
VL: HIV viral load
