# Correction to: An outbreak of HIV infection among people who inject drugs in northeastern Massachusetts: findings and lessons learned from a medical record review

**DOI:** 10.1186/s12889-022-12808-7

**Published:** 2022-03-01

**Authors:** Liisa M. Randall, Sharoda Dasgupta, Jeanne Day, Alfred DeMaria, Joseph Musolino, Betsey John, Kevin Cranston, Kate Buchacz

**Affiliations:** 1 Massachusetts Department of Public Health, Bureau of Infectious Disease and Laboratory Sciences, 305 South Street, Jamaica Plain, MA 02130-3515 USA; 2 Division of HIV/AIDS Prevention, Centers for Disease Control and Prevention, Atlanta, GA USA; 3 JSI Research and Training Institute, Inc., Boston, MA USA

**Correction to: BMC Public Health 22, 257 (2022)**

**https://doi.org/10.1186/s12889-022-12604-3**

During the publication process of the original article several citation errors were introduced. The correct and incorrect information is shown below. The original article has been updated. The publisher apologizes to the authors & readers for the inconvenience caused.

**Table:** 

**Incorrect**	**Correct**
We found evidence of marked engagement in drug and alcohol treatment, but possible suboptimal use of MAT. We documented high levels of ART prescription and viral suppression was higher than the overall rate in Massachusetts (65%), but lower than the state’s rate of viral suppression among patients retained in care (88%) [**23**].	We found evidence of marked engagement in drug and alcohol treatment, but possible suboptimal use of MAT. We documented high levels of ART prescription and viral suppression was higher than the overall rate in Massachusetts (65%), but lower than the state’s rate of viral suppression among patients retained in care (88%) [**22**].
The effectiveness of SSPs can be enhanced through provision of testing for HIV and other infections as well as other prevention services such as vaccinations and HIV PrEP [**24**].	The effectiveness of SSPs can be enhanced through provision of testing for HIV and other infections as well as other prevention services such as vaccinations and HIV PrEP [**23**].
Our findings demonstrate that medical record review in the context of an outbreak investigation can benefit health jurisdictions without integrated infectious disease management systems by providing data on co-occurring conditions or clinical indications of early diagnosis. Medical records data can also suggest structural barriers to accessing services, missed opportunities for public health intervention and can facilitate exchange of information between surveillance and field staff needed to identify possible factors for transmission [**22, 25**].	Our findings demonstrate that medical record review in the context of an outbreak investigation can benefit health jurisdictions without integrated infectious disease management systems by providing data on co-occurring conditions or clinical indications of early diagnosis. Medical records data can also suggest structural barriers to accessing services, missed opportunities for public health intervention and can facilitate exchange of information between surveillance and field staff needed to identify possible factors for transmission [**24, 25**].

